# Immune checkpoint inhibitor‐related adverse events in lung cancer: Real‐world incidence and management practices of 1905 patients in China

**DOI:** 10.1111/1759-7714.14274

**Published:** 2021-12-21

**Authors:** Yuequan Shi, Jian Fang, Chengzhi Zhou, Anwen Liu, Yan Wang, Qingwei Meng, Cuimin Ding, Bin Ai, Yangchun Gu, Yu Yao, Hong Sun, Hui Guo, Cuiying Zhang, Xia Song, Junling Li, Bei Xu, Zhiqiang Han, Meijun Song, Tingyu Tang, Peifeng Chen, Hongmin Lu, Yongjie Shui, Guangyuan Lou, Dongming Zhang, Jia Liu, Xiaoyan Liu, Xiangning Liu, Xiaoxing Gao, Qing Zhou, Minjiang Chen, Jing Zhao, Wei Zhong, Yan Xu, Mengzhao Wang

**Affiliations:** ^1^ Department of Respiratory and Critical Care Medicine, Peking Union Medical College Hospital Chinese Academy of Medical Sciences & Peking Union Medical College Beijing China; ^2^ Department of Thoracic Oncology II, Key Laboratory of Carcinogenesis and Translational Research (Ministry of Education/Beijing) Peking University Cancer Hospital and Institute Beijing China; ^3^ State Key Laboratory of Respiratory Disease National Clinical Research Center of Respiratory Disease, Guangzhou Institute of Respiratory Health, the First Affiliated Hospital of Guangzhou Medical University, Guangzhou Medical University Guangzhou China; ^4^ Department of Oncology The Second Affiliated Hospital of Nanchang University, Nanchang University Nanchang China; ^5^ Department of Medical Oncology, National Cancer Center, National Clinical Research Center for Cancer, Cancer Hospital Chinese Academy of Medical Sciences & Peking Union Medical College Beijing China; ^6^ Department of Medical Oncology Harbin Medical University Cancer Hospital Harbin China; ^7^ Department of Respiratory Medicine The Fourth Hospital of Hebei Medical University Shijiazhuang China; ^8^ Department of Medical Oncology, Beijing Hospital, National Center of Gerontology, Institute of Geriatric Medicine, Chinese Academy of Medical Sciences Beijing China; ^9^ Department of Medical Oncology and Radiation Sickness Peking University Third Hospital Beijing China; ^10^ Department of Medical Oncology The First Affiliated Hospital of Xi'an Jiaotong University Xi'an China; ^11^ Cancer Center People's Hospital Huhehot China; ^12^ Department of Respiratory Medicine Shanxi Provincial Cancer Hospital Taiyuan China; ^13^ Department of Medical Oncology Zhongshan Hospital Fudan University Shanghai China; ^14^ Department of Respiratory and Critical Care Medicine Quzhou People's Hospital Zhejiang China; ^15^ Department of Respiratory Medicine Zhejiang Provincial People's Hospital Zhejiang China; ^16^ Department of Respiratory Medicine, Affiliated Zhejiang Hospital Zhejiang University School of Medicine Zhejiang China; ^17^ Department of Respiratory Medicine and Critical Care Medicine Zhuji People's Hospital Zhejiang China; ^18^ Department of Oncology, Renji Hospital Affiliated to Shanghai Jiaotong University School of Medicine Shanghai China; ^19^ Department of Radiation Oncology, The Second Affiliated Hospital Zhejiang University School of Medicine Hangzhou China; ^20^ Department of Medical Oncology The Cancer Hospital of the University of Chinese Academy of Sciences (Zhejiang Cancer Hospital) Hangzhou China

**Keywords:** advanced lung cancer, immune checkpoint inhibitors, immune‐related adverse events, real‐world data

## Abstract

**Background:**

Immune checkpoint inhibitors (ICIs) are the standard treatment for advanced lung cancer, but immune‐related adverse events (irAEs) remain poorly understood, especially in a real‐world setting.

**Methods:**

A multicenter observational study was conducted. Medical records of lung cancer patients treated with ICIs at 26 hospitals from January 1, 2015, to February 28, 2021, were retrieved. Types of ICIs included antiprogrammed cell death 1 or antiprogrammed cell death ligand 1 (PD‐L1) monotherapy, anticytotoxic T‐lymphocyte antigen‐4 monotherapy, or combination therapy.

**Results:**

In total, 1905 patients with advanced lung cancer were evaluated. The median age was 63 (range 28–87) years, and the male/female ratio was 3.1:1 (1442/463). The primary histological subtype was adenocarcinoma (915). A total of 26.9% (512/1905) of the patients developed 671 irAEs, and 5.8% (110/1905) developed 120 grade 3–5 irAEs. Median duration from ICI initiation to irAEs onset was 56 (range 0–1160) days. The most common irAEs were thyroid dysfunction (7.2%, 138/1905), pneumonitis (6.5%, 124/1905), and dermatological toxicities (6.0%, 115/1905). A total of 162 irAEs were treated with steroids and 11 irAEs led to death. Patients with positive PD‐L1 expression (≥1%) and who received first‐line ICI treatment developed more irAEs. Patients who developed irAEs had a better disease control rate (DCR, 71.3% [365/512] vs. 56.0% [780/1145]; *p* < 0.001).

**Conclusions:**

The incidence rate of irAEs was 26.9% in a real‐world setting. IrAEs might be related to a better DCR, but clinicians should be more aware of irAE recognition and management in clinical practice.

## Graphical abstract

This was a multicenter observational study of real‐world treatment status immune checkpoint inhibitors (ICIs) and incidence of immune‐related adverse events (irAEs). A total of 512 (26.9%) of 1905 patients developed 671 any grade irAEs and 110 (5.8%) patients developed 120 grades 3–5 irAEs. Positive programmed cell death‐ligand 1 expression, first‐line ICI treatment and sustained disease control were observed more in patients with irAEs.

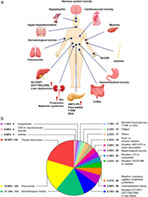

## INTRODUCTION

Immune checkpoint inhibitors (ICIs) have substantially improved clinical outcomes in many types of cancer and are increasingly being used in early disease settings, including advanced lung cancer.^1^ Response to treatment occurs in a substantial fraction of patients and is frequently durable. The Food and Drug Administration approved the antiprogrammed cell death‐1 (PD‐1) antibody, pembrolizumab in combination with chemotherapy, as a first‐line therapy for metastatic non‐small cell lung cancer (NSCLC), irrespective of programmed cell death ligand‐1 (PD‐L1) status in July 2018 based on the results reported by the Keynote‐189^2^ and Keynote‐407 studies.^3^ Subsequently, pembrolizumab was approved for metastatic NSCLC by the National Medical Products Administration of China on March 28, 2019. Other anti‐PD‐1/PD‐L1 antibodies, including camrelizumab, sintilimab, and tislelizumab, have also been approved in China. Currently, PD‐1/ PD‐L1 monotherapy or PD‐1/PD‐L1‐combined chemotherapy has become the first‐line standard treatment for advanced lung cancer according to the status of PD‐L1 expression.

As with other treatments, use of ICIs has been associated with immune‐related adverse events (irAEs) that are potentially severe, or even fatal.^4^, ^5^ The incidence of irAEs have been reported to vary between 24% and 38% in patients with advanced lung cancer treated with ICI‐based therapy.^6^, ^7^, ^8^, ^9^, ^10^, ^11^ However, the above reported data were from randomized clinical trials (RCTs), which did not report irAEs in a real‐world setting. The special population for ICI treatment includes patients affected by chronic viral infection or with pre‐existent autoimmune diseases (AIDs), patients aged over 75 years, or those with an Eastern Cooperative Oncology Group (ECOG) performance status (PS) score of 2–3. Therefore, it is crucial to obtain real‐world information about the safety profile of ICIs in patients. Furthermore, irAE management has rarely been reported.

Hence, we conducted this observational study to identify the incidence, spectrum, clinical characteristics, and management practices of irAEs in a real‐world setting in Chinese patients with advanced lung cancer.

## METHODS

### Study population

This multicenter observational study aimed to evaluate the safety of ICIs and investigate the status of irAE management practices in a real‐world setting in China. We enrolled patients who were (1) >18 years, (2) had pathologically confirmed stage III–IV lung cancer including non‐small cell lung cancer (NSCLC) and small cell lung cancer (SCLC) and (3) treated with ICI monotherapy or ICI‐based combination therapy for at least one dose, from 26 hospitals across 10 provinces in China between January 1, 2015, and February 28, 2021.

### Data collection and recognition of irAEs

Demographic and clinical characteristics of patients were collected, including age, sex, comorbidities, clinical stage, number of metastatic sites, tumor histology type, ECOG PS status, driving gene mutations, PD‐L1 expression status, treatment type, treatment line of ICIs, ICI duration, and disease control rate (DCR). Response assessment was performed according to the Response Evaluation Criteria in Solid Tumors version 1.1 (RECIST 1.1) by computed tomography scans every 6 to 8 weeks after the administration of the first dose of ICIs by the investigator.^12^

The definition of irAEs was based on (1) pathological evidence of irAE, (2) multidisciplinary adjudication including two or more oncologists, or (3) clinical improvement with an irAE‐based treatment.^13^, ^14^, ^15^, ^16^ Data was collected on the management practices of irAEs, including use and duration of steroids, and outcome of irAEs. Immune toxicity‐related discontinuation of ICIs and rechallenge with ICIs were also collected.

The procedures followed were in accordance with the principles of Good Clinical Practice and the Declaration of Helsinki. The study was approved by the local ethics committee on human experimentation (Peking Union Medical College Hospital, Internal Review Board protocol number SK‐1315, approved on August 31, 2020).

### Statistical analysis

Descriptive statistics were used to summarize the cohorts' medical histories and clinical parameters. Counts and percentages were produced for categorical variables, whereas the mean ± standard deviation (SD) or median (interquartile range [IQR]) were computed for continuous variables. The chi‐squared and Mann–Whitney U tests were used to compare the demographic characteristics between patients without irAEs and those with irAEs. All statistical analyses were performed using the SPSS software (version 26.0; SPSS). The figures were developed using GraphPad Prism 8.0.

## RESULTS

### Patient characteristics

A total of 1905 patients with advanced lung cancer were enrolled in this study. The median age was 63 years (range 28–87) years. Many of the patients had NSCLC (89.9%, 1712/1905), where 48.0% (915/1712) had adenocarcinoma, and 34.0% (647/1712) had squamous cell carcinoma. A total of 471 patients had a positive driver mutation record, and the leading driving mutations were epidermal growth factor receptor (EGFR) 19/21 (*n* = 163) and *KRAS* (*n* = 132). PD‐L1 expression was determined from histological specimens in 441 (23.1%) cases, and 283 (64.2%, 283/441) showed positive PD‐L1 results (PD‐L1 ≥ 1%). A total of 1488 patients were diagnosed with metastatic disease, with 208 patients developing liver metastases and 330 developing brain metastases. The clinical features of the 1905 patients with advanced lung cancer treated with ICI‐based treatment are shown in Table 1.

**TABLE 1:** Clinical features of the 1905 patients with advanced lung cancer treated with ICI‐based treatment

Characteristic	Total	Without irAEs	With irAEs	*p*‐value
	*N* = 1905	*N* = 1393	*N* = 512	
Age, median (IQR), year	63 (56.25, 68)	63 (56, 68)	64 (58, 69)	0.902
Age				0.902
>75	128 (6.7%)	93 (6.7%)	35 (6.8%)	
<75	1777 (93.3%)	1300 (93.3%)	477 (93.2%)	
Sex				0.063
Female	463 (24.3%)	354 (25.4%)	109 (21.3%)	
Male	1442 (75.7%)	1039 (74.6%)	403 (78.7%)	
History of Interstitial pneumonitis				1.000
Yes	14 (0.7%)	10 (0.7%)	4 (0.8%)	
No	1891 (99.3%)	1383 (99.3%)	508 (99.2%)	
History of autoimmune disease				0.353
Yes	13 (0.7%)	8 (0.6%)	5 (1.0%)	
No	1892 (99.3%)	1385 (99.4%)	507 (99.0%)	
History of chronic viral infection				0.819
Yes	35 (1.8%)	25 (1.8%)	10 (2.0%)	
No	1870 (98.2%)	1368 (98.2%)	502 (98.0%)	
Clinical stage				0.795
III	417 (21.9%)	307 (22.0%)	110 (21.5%)	
IV	1488 (78.1%)	1086 (78.0%)	402 (78.5%)	
Number of metastatic sites				
>2	324 (17.0%)	254 (18.2%)	70 (13.7%)	0.024*
≤2	1581 (83.0%)	1139 (81.8%)	442 (86.3%)	
Histologic types				0.076
Non‐small cell lung cancer	1709 (89.7%)	1240 (89.0%)	469 (91.6%)	
Adenocarcinoma	915 (48.0%)	667 (47.9%)	248 (48.4%)	
Squamous cell carcinoma	647 (34.0%)	464 (33.3%)	183 (35.7%)	
Large cell carcinoma	34 (1.8%)	20 (1.4%)	14 (2.7%)	
Other	113 (5.9%)	89 (6.4%)	24 (4.7%)	
Small cell lung cancer	196 (10.3%)	153 (11.0%)	43 (8.4%)	
ECOG performance status				0.480
0–1	1755 (92.1%)	1277 (91.7%)	478 (93.4%)	
2–3	150 (7.9%)	116 (8.3%)	34 (6.6%)	
*EGFR* 19/21 mutation				0.024*
Positive	163 (8.6%)	127 (9.1%)	36 (7.0%)	
Negative	670 (35.2%)	466 (33.5%)	204 (39.8%)	
Not assessed	1072 (56.3%)	800 (57.4%)	272 (53.1%)	
*KRAS* mutation				0.900
Positive	132 (6.9%)	96 (6.9%)	36 (7.0%)	
Negative	306 (16.1%)	227 (16.3%)	79 (15.4%)	
Not assessed	1467 (77.0%)	1070 (76.8%)	397 (77.5%)	
PD‐L1 expression status				0.000*
Positive	283 (14.9%)	176 (12.6%)	107 (20.9%)	
Negative	158 (8.3%)	113 (8.1%)	45 (8.8%)	
Not assessed	1464 (76.9%)	1104 (79.3%)	360 (70.3%)	
Treatment line of ICI				0.001*
First line	1056 (55.4%)	745 (53.5%)	311 (60.7%)	
Second line	497 (26.1%)	365 (26.2%)	132 (25.8%)	
Third or more	352 (18.5%)	283 (20.3%)	69 (13.5%)	
Treatment pattern				0.130
Concurrent with chemotherapy	1162 (61.0%)	864 (62.0%)	298 (58.2%)	
ICI only	743 (39.0%)	529 (38.0%)	214 (41.8%)	
ICI duration, median (IQR), cycle	5 (3–10)	5 (2–8)	6 (4–12)	0.000*
Best treatment response				0.000*
CR/PR/SD	1145 (60.1%)	780 (56.0%)	365 (71.3%)	
PD	163 (8.6%)	136 (9.8%)	27 (5.3%)	
NA	597 (31.3%)	477 (34.2%)	120 (23.4%)	

Abbreviations: ICI, immune checkpoint inhibitor; irAEs, immune‐related adverse events; IQR, interquartile range; ECOG, Eastern Cooperative Oncology Group; EGFR, epidermal growth factor receptor; PD‐L1, programmed cell death‐ligand 1; CR, complete response; PR, partial response; SD, stable disease; PD, progressive disease. *, means that the *p* value is statistically significant.

**FIGURE 1:**
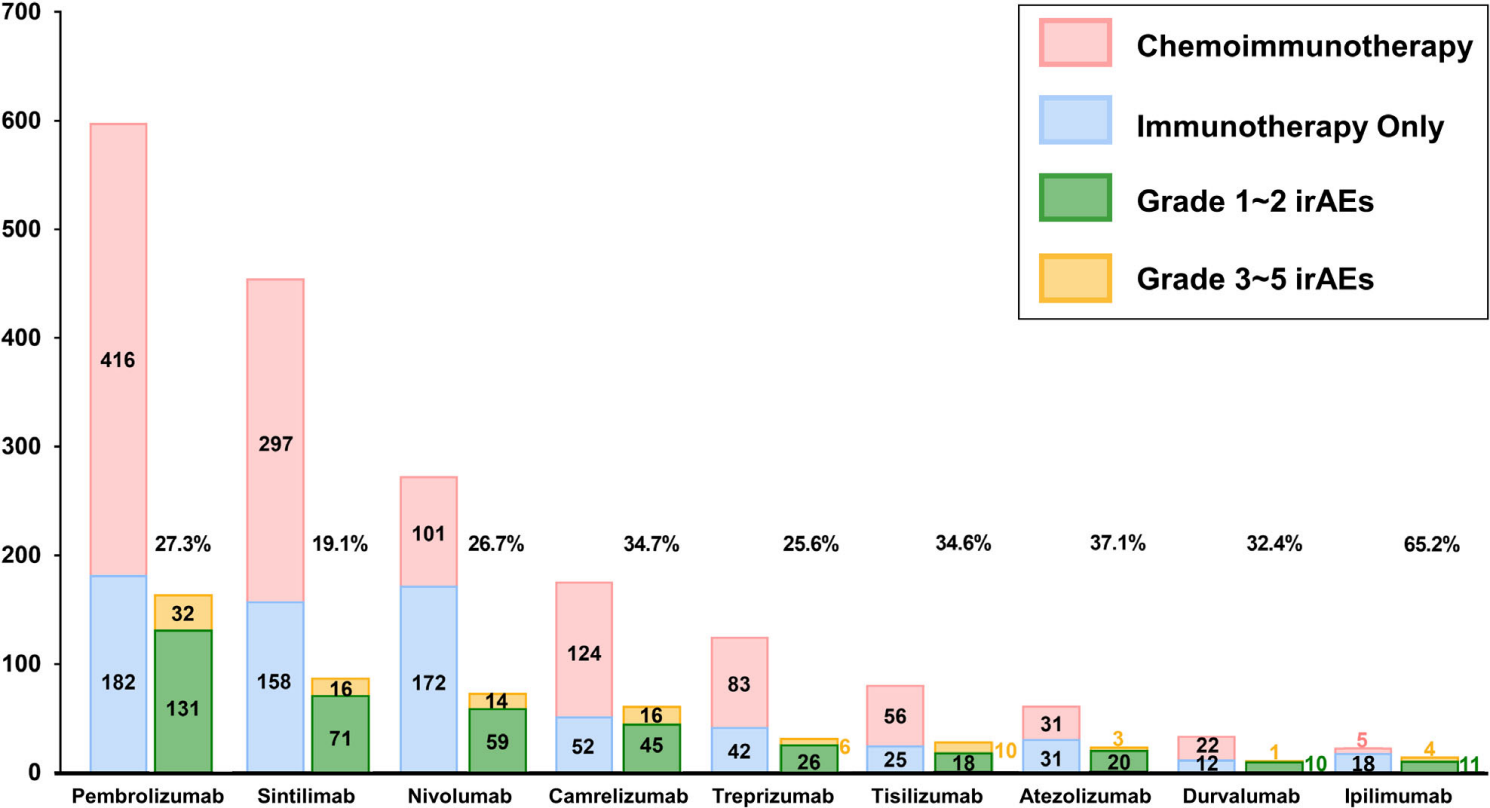
Treatment patterns of immune checkpoint inhibitors and incidence of immune‐related adverse events according to ICI types. irAEs, immune‐related adverse events

The median number of ICI cycles received was five (IQR, 3–10). The most widely used ICI was pembrolizumab (*n* = 598), followed by sintilimab (*n* = 455), nivolumab (*n* = 273), and camrelizumab (*n* = 176). The treatment patterns and types of ICIs are shown in Figure 1. The mean treatment course of pembrolizumab was 5.7 months, followed by 4.3 months for sintilimab, 4.1 months for nivolumab, 3.6 months for camrelizumab and 3.9 months for treprizumab. The treatment durations and the mean incidence rate of irAEs per month according to ICIs type are listed in Table 2. A total of 55.4% (1056/1905) of the patients received ICIs as first‐line therapy, and 497 (26.1%) patients received ICIs as second‐line therapy. More than half of the patients (61.0%, 1162/1905) received ICI‐combined chemotherapy, paclitaxel being mostly used as the cytotoxic partner drug (*n* = 494). Only 23 patients received cytotoxic T lymphocyte antigen‐4 (CTLA‐4) antibody plus nivolumab or durvalumab.

**TABLE 2:** Treatment duration and irAEs incidence per treatment months according to ICI types

	Number of patients	Total duration of ICI exposure (months)	Mean duration of ICI exposure (months)	Number of patients who developed irAEs	Mean incidence of irAEs /months
Pembrolizumab	598	3409	5.7	163	4.8%
Sintilimab	455	1957	4.3	87	4.4%
Nivolumab	273	1119	4.1	73	6.5%
Camrelizumab	176	634	3.6	61	9.6%
Treprizumab	125	488	3.9	32	6.6%
Tisilizumab	81	599	7.4	28	4.7%
Atezolizumab	62	329	5.3	23	7.0%
Durvalumab	34	211	6.2	11	5.2%

Abbreviations: ICI, immune checkpoint inhibitor; irAEs, immune‐related adverse events.

### Treatment response

A total of 1308 patients were available with response assessment records. A total of 518 patients experienced the best overall response of partial response (PR), and 627 patients experienced stable disease (SD).

In patients with squamous NSCLC who received first‐line therapy, the objective response rate (ORR) was 54.5% in patients receiving ICI‐combined chemotherapy, and 37.3% in patients receiving ICI monotherapy (*p* = 0.019). The DCRs were 93.6% and 93.2%, respectively (*p* = 0.908).

As for patients with nonsquamous NSCLC who were not positive for *EGFR* 19/21 mutation and received first‐line treatment, the ORR was 53.4% in patients who received ICI‐combined chemotherapy, and 40.2% in patients receiving ICI monotherapy (*p* = 0.027). The DCRs were 95.2% and 87.6%, respectively (*p* = 0.013).

In patients with nonsquamous NSCLC who were positive for *EGFR* 19/21 mutations and failed targeted first‐line therapy, the ORR of patients who received ICI‐combined chemotherapy was 40.6%, while the ORR of patients who received ICI monotherapy was 27.3% (*p* = 0.494). The DCR was 100.0% and 54.5%, respectively (*p* = 0.000).

For patients with SCLC who received first‐line ICI‐combined chemotherapy (*n* = 66), the ORR was 74.2% (49 PR, 49/66), and the DCR was 90.9% (49 PR, 11 SD, 60/66). The treatment responses are presented in Table 3.

**TABLE 3:** Treatment response of 1308 patients

	Total *(n* = 1308)	Chemo‐immunotherapy (*n* = 785)	Immunotherapy only (*n* = 523)	*p*‐value
Squamous cell carcinoma	*N* = 455	*N* = 284	*N* = 171	
First‐line	*N* = 279	*N* = 220	*N* = 59	
ORR	51.6% (144)	54.5% (120)	37.3% (22)	0.019*
DCR	94.3% (263)	93.6% (206)	93.2% (55)	0.908
Second‐line	*N* = 127	*N* = 50	*N* = 77	
ORR	33.9% (43)	42.0% (21)	28.6% (22)	0.118
DCR	84.3% (107)	88.0% (44)	81.8% (63)	0.350
Nonsquamous cell carcinoma	*N* = 735	*N* = 411	*N* = 324	
Without positive *EGFR* mutation	*N* = 610	*N* = 329	*N* = 281	
First‐line	*N* = 346	*N* = 249	*N* = 97	
ORR	49.7% (172)	53.4% (133)	40.2% (39)	0.027*
DCR	93.1% (332)	95.2% (237)	87.6% (85)	0.013*
Second‐line	*N* = 167	*N* = 51	*N* = 116	
ORR	25.7% (43)	31.4% (16)	23.3% (27)	0.270
DCR	86.8% (145)	90.2% (46)	85.3% (99)	0.393
With positive EGFR 19/21 mutation who failed targeted therapy	*N* = 125	*N* = 82	*N* = 43	
Second‐line^a^	*N* = 43	*N* = 32	*N* = 11	
ORR	37.3% (16)	40.6% (13)	27.3% (3)	0.494
DCR	65.1% (38)	100% (32)	54.5% (6)	0.000*

Abbreviations: ORR, overall response rate; DCR, disease control rate; EGFR, epidermal growth factor receptor; ORR, overall response rate.

a Second line refers to patients who received TKI as first‐line therapy. *, means that the *p* value is statistically significant.

### Incidence and spectrum of irAEs

**FIGURE 2:**
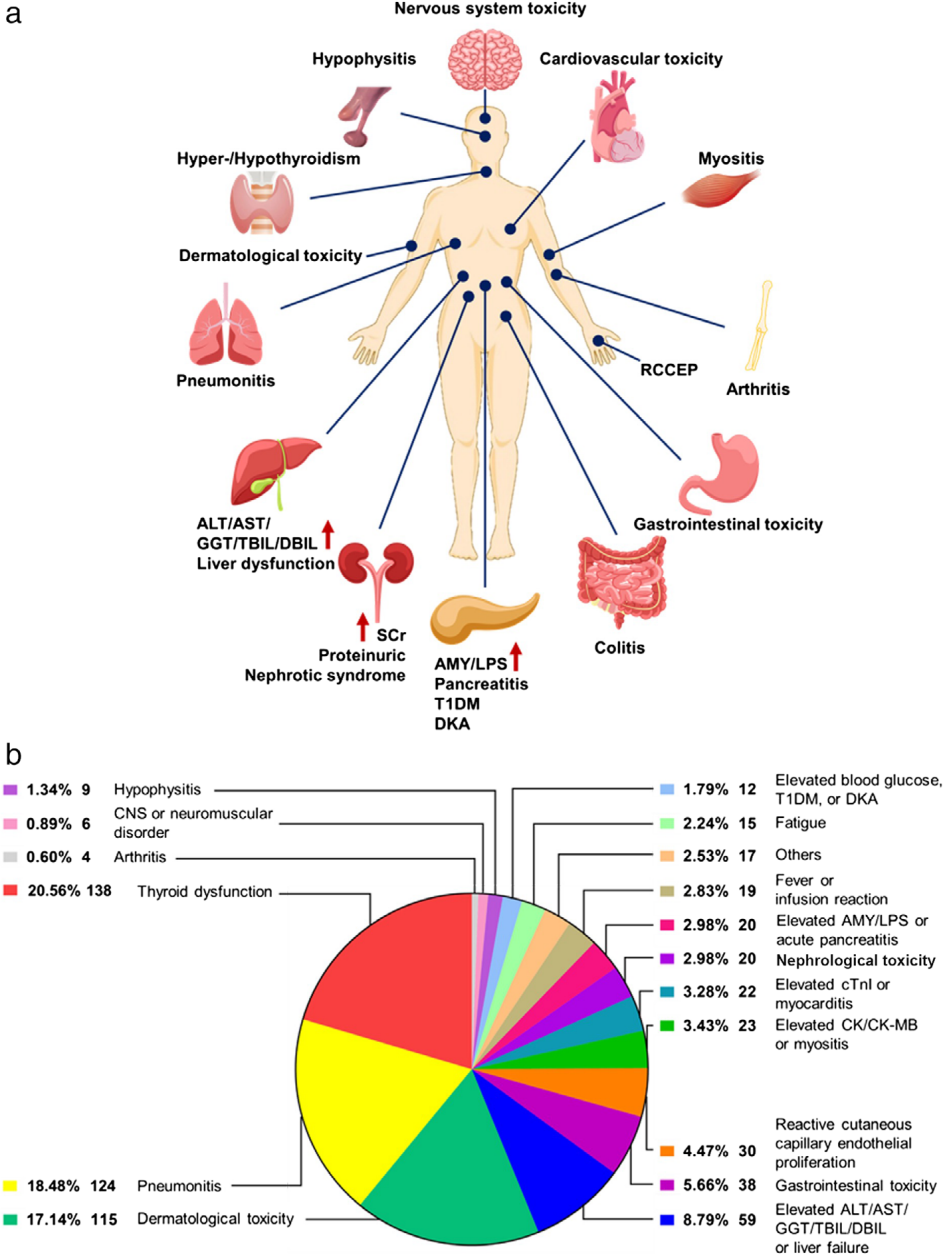
The organ distribution and spectrum of immune‐related adverse events. RCCEP, reactive capillary endothelial proliferation; ALT, alanine aminotransferase; AST, aspartate aminotransferase; GGT, gamma‐glutamyl transpeptidase; TBIL, total bilirubin; DBIL, direct bilirubin; SCr, serum creatinine; AMY, amylase; LPS, lipase; T1DM, type 1 diabetes; DKA, diabetic ketoacidosis; CNS, central nervous system; cTnI, cardiac troponin I; CK, creatine kinase; CK‐MB, creatine kinase isoenzymes

A total of 671 irAEs were observed in 26.9% of patients (512/1905). The most common overall organ system immune‐related toxicities were the endocrine system (8.3%, 159/1905), pulmonary (6.7%, 124/1905), and skin (6.0%, 115/1905). For the subset of patients reported as having endocrine toxicities, the majority were thyroid dysfunction (7.2%, *n* = 138), including 108 hypothyroidism and 30 hyperthyroidism patients, followed by type I diabetes mellitus (including diabetic ketoacidosis [DKA], *n* = 12) and hypophysitis (*n* = 9). All patients who developed pulmonary toxicity were identified as having pneumonitis (6.7%, *n* = 124). For patients with dermatological toxicities, the majority were rash (*n* = 52), pruritus (*n* = 24), or both (*n* = 8). The incidence of grade 3–5 irAEs was 5.8% (110/1905), with the most common grades 3–5 irAEs being pneumonitis (1.8%, 35/1905), followed by dermatological toxicities (1.2%, 22/1905) and increased alanine aminotransferase (ALT, 0.8%, 16/1905). Eleven irAEs led to death, including nine from pneumonitis, one from liver failure, and one from myocarditis. Other irAEs (*n* = 17) included thrombocytopenia (*n* = 3), palpitation (*n* = 2), anemia (*n* = 2), hypoproteinemia (*n* = 1), elevated eosinophils (*n* = 2), elevated lactic dehydrogenase (*n* = 2), xerophthalmia (*n* = 1), cough (*n* = 1), drug‐induced sarcoidosis‐like reaction (*n* = 1), cholangitis (*n* = 1), and sialadenitis of the submandibular gland (*n* = 1). The spectrum of irAEs of our study is shown in Figure 2, and the organ distribution of grade 1–2 irAEs and grade 3–5 irAEs is shown in Table 4.

The incidence of irAEs according to the different ICIs is shown in Figure 1. Of the patients who received pembrolizumab (*n* = 598), 27.3% (*n* = 163) developed irAEs of any grade; this was followed by 19.1% (*n* = 87) of the patients who received sintilimab (*n* = 455), 26.7% (*n* = 73) of the patients who received nivolumab (*n* = 273), and 34.7% (*n* = 61) of the patients who received camrelizumab (*n* = 176) developed irAEs (Figure 1).

**TABLE 4:** Organ distribution of grade 1–2 irAEs and grade 3–5 irAEs

irAEs	All irAEs	Grade 1–2 irAEs	Grade 3–5 irAEs
	*N* = 671	*N* = 556	*N* = 115
Thyroid dysfunction	138	136 (98.6%)	2 (1.4%)
Pneumonitis	124	89 (71.8%)	35 (28.2%)
Dermatological toxicity	115	93 (80.9%)	22 (19.1%)
Elevated ALT/AST/GGT/TBIL/DBIL or liver failure	59	43 (72.9%)	16 (27.1%)
Gastrointestinal toxicity	38	28 (73.7%)	10 (26.3%)
Reactive cutaneous capillary endothelial proliferation	30	30 (100.0%)	0
Elevated CK/CK‐MB or myositis	23	20 (87.0%)	3 (13.0%)
Elevated cTnI or myocarditis	22	13 (59.1%)	9 (40.9%)
Elevated AMY/LPS or acute pancreatitis	20	15 (75.0%)	5 (25.0%)
Nephrological toxicity	20	17 (85.0%)	3 (15.0%)
Fever or infusion reaction	19	18 (94.7%)	1 (5.3%)
Fatigue	15	15 (100.0%)	0
Elevated blood glucose, type I diabetes or DKA	12	7 (58.3%)	5 (41.7%)
Hypophysitis	9	4 (44.4%)	5 (55.6%)
CNS or neuromuscular disorder	6	5 (83.3%)	1 (16.7%)
Arthritis	4	4 (100.0%)	0
Others	17	14 (82.4%)	3 (17.6%)

Abbreviations: irAEs, immune‐related adverse events; ALT, alanine aminotransferase; AST, aspartate aminotransferase; GGT, gamma‐glutamyl Transpeptidase; TBIL, total bilirubin; DBIL, direct bilirubin; CK, creatine kinase; CK‐MB, creatine kinase isoenzymes; cTnI, cardiac troponin I; AMY, amylase; LPS, lipase; DKA, diabetic ketoacidosis; CNS, central nervous system.

Among patients with irAEs (*n* = 512), 20.7% (394/1905) developed single system irAEs, and 6.2% (118/1905) developed multisystem irAEs (277 irAEs) with a maximum of four organ systems. Most patients with multisystem irAEs had two irAEs (4.3%, 81/1905). The most common irAEs were dermatitis (10.8%, 30/277) and thyroid dysfunction (9.0%, 25/277). The most common multisystem irAE patterns were dermatitis and thyroiditis (10.2%, 12/118), thyroiditis and pneumonitis (4.2%, 5/118), and pneumonitis and hepatitis (3.4%, 4/118).

**FIGURE 3:**
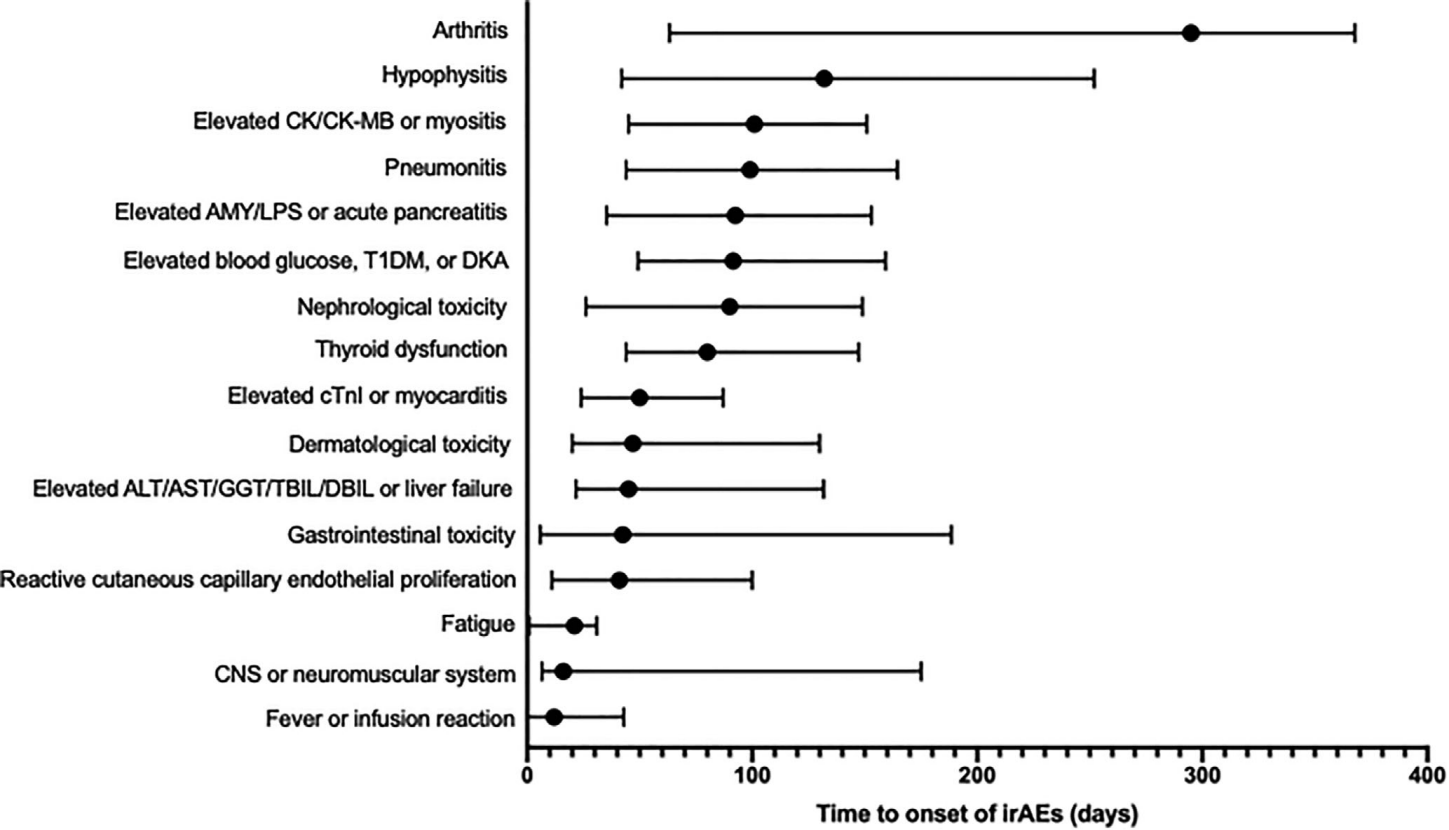
Duration between ICI initiation and onset of irAEs. The durations between ICI initiation and onset of irAEs are ploted with the median (the black dot) with interquartile range. The median duration for each irAEs are listed here: fever or infusion reaction, 12 days; CNS or neuromuscular system, 16 days; fatigue, 21 days; reactive cutaneous capillary endothelial proliferation, 41 days; gastrointestinal toxicity, 42.5 days; elevated ALT/AST/GGT/TBIL/DBIL or liver failure, 45 days; dermatological toxicity, 47 days; elevated cTnI or myocarditis, 50 days; thyroid dysfunction, 80 days; nephrological toxicity, 90 days; elevated blood glucose, T1DM, or DKA, 91.5 days; elevated AMY/LPS or acute pancreatitis, 92.5 days; pneumonitis, 99 days; elevated CK/CK‐MB or myositis, 101 days; hypophysitis, 132 days; and arthritis, 295 days. Abbreviations: ICI, immune checkpoint inhibitor; irAEs, immune‐related adverse events; CNS, central nervous system; ALT, alanine aminotransferase; AST, aspartate aminotransferase; GGT, gamma‐glutamyl transpeptidase; TBIL, total bilirubin; DBIL, direct bilirubin; cTnI, cardiac troponin I; T1DM, type 1 diabetes; DKA, diabetic ketoacidosis; AMY, amylase; LPS, lipase; CK, creatine kinase; CK‐MB, creatine kinase isoenzymes

The median time from ICI initiation to the onset of the first irAE was 56 (range 0–1160) days, and to the second irAE was 124 days (range 6–879 days). There was no significant difference between the median time from ICI initiation to the onset of grade 1–2 irAEs (62, range 0–1239 days) and grade 3–5 irAEs (71, range 3–726 days; *p* = 0.151). The median time to onset of irAEs according to organ involvement is shown in Figure 3.

### Management practices and clinical outcomes of irAEs

Of the 671 immune‐related toxicities, 24.1% (*n* = 162) were treated with steroids, including pneumonitis (*n* = 86), dermatitis (*n* = 16), hepatitis (*n* = 14), myocarditis (*n* = 10), colitis (*n* = 9), hypophysitis (*n* = 4), myositis (*n* = 4), pancreatitis (*n* = 3), encephalitis (*n* = 2), arthralgia (*n* = 2), and others (*n* = 12). Patients who developed endocrine system disorder and received hormone replacement treatment were not considered as treated with steroids. The median treatment course of steroids was 49 (range 1–300) days. Most patients improved after steroid therapy, and seven patients developed aggravated pneumonitis during steroid withdrawal. Intravenous immunoglobulin was used in 101 patients. Six patients were treated with an immunosuppressive or biological agent, including etanercept for grade 4 Stevens–Johnson syndrome, mycophenolate mofetil for grade 3 hepatitis, tocilizumab for grade 3 hepatitis, cyclosporine for grade 3 pneumonitis, rituximab for grade 3 proteinuria caused by membranous nephropathy, and tripterygium wilfordii for grade 3 eosinophilic dermatosis. A total of 178 irAEs led to discontinuation of the ICI treatment. One patient developed colitis after receiving ipilimumab plus nivolumab and continued with only nivolumab after relief of colitis.

Clinicians attempted to rechallenge ICI treatment after 51 irAEs occurred. The leading irAEs were pneumonitis (*n* = 22), colitis (*n* = 9), and DKA (*n* = 5). One patient with pneumonitis and one patient with an infusion reaction developed relapse of the former irAEs after ICI rechallenge, and ICI treatment was then permanently discontinued.

### Safety of ICIs in special population

Among the 1905 patients enrolled in this study, 13 patients had pre‐existing AID, including rheumatoid arthritis (*n* = 4), psoriasis (*n* = 3), Sjogren’s syndrome (*n* = 2), polymyalgia rheumatica (*n* = 1), bullous pemphigoid (*n* = 1), scleroderma (*n* = 1), and Guillain‐Barre syndrome (*n* = 1). Fourteen patients had comorbid mild interstitial pneumonitis prior to ICI treatment. Thirty‐five patients had chronic viral infection, including 14 patients who were hepatitis B virus carriers. There were seven patients with a history of hepatitis A (*n* = 2), B (*n* = 3), and C (*n* = 2) virus infection, which was relieved before ICI treatment. Two patients were positive for Treponema pallidum, and 12 patients had a history of HBV infection, but the detailed information was scant. A total of 128 patients were > 75 years old, and 150 patients were treated with ECOG PS 2–3 pre‐ICI‐treatment.

The comparison of clinical characteristics between patients with and without irAEs showed that patients who developed irAEs were more likely to have fewer metastatic sites (without irAEs vs. with irAEs; 18.2% vs. 13.7%, *p* = 0.024), negative *EGFR* 19/21 mutations (33.5% vs. 39.8%, *p* = 0.024), positive PD‐L1 expression status (12.6% vs 20.9%, *p* < 0.001), first‐line ICIs treatment (53.5% vs. 60.7%, *p* = 0.001), and sustained disease control (CR + PR + SD, 56.0% vs 71.3%, *p* = 0.001). Median ICI duration was longer in patients who developed irAEs (median cycles, 5 vs. 6, *p* < 0.001). No significant differences in the incidence of irAEs were recorded in patients with comorbid diseases, age > 75 years, or poor ECOG PS status. The results are listed in Table 1.

## DISCUSSION

In this study, we report the status of ICI treatment, clinical characteristics, and management practices of irAEs in the largest cohort studied to date in real‐world patients with advanced lung cancer in China. We observed an irAE incidence rate of 26.9%, with an incidence rate of grade 3–5 irAEs of 5.8%. This result is consistent with the data from prospective clinical trials reported in the literature.^6^, ^7^, ^8^, ^9^, ^10^, ^11^ Moreover, we found a relationship between positive PD‐L1 expression, longer ICI treatment duration, better DCR, and incidence of irAEs. No significantly higher incidence of irAEs was observed in this population.

Our study found that patients with positive PD‐L1 expression had a significantly higher incidence of irAEs. A meta‐analysis of 6696 patients reported that higher PD‐L1 expression was significantly associated with the development of irAEs in multivariate analysis.^17^ PD‐L1 expression refers to the membrane expression of PD‐L1 by tumor cells, which was presumed to be a predictive biomarker for the patient response early in the use of anti‐PD‐(L)1 agents, which has been proven by several clinical trials.^2^, ^18^, ^19^, ^20^ Due to the relationship between the incidence of irAEs and better treatment response, it is essential to understand the relationship between high PD‐L1 expression and increased irAE incidence.

We also observed that patients who developed irAEs had longer ICI durations and better DCRs than patients without irAEs. This result is in accordance with the published data. Across disease sites, including lung cancer and other solid tumors, patients who experience irAEs while on therapy with anti‐PD‐1 and anti‐PD‐L1 antibodies have been documented to experience improved outcomes as measured by ORR, progression‐free survival (PFS), and overall survival (OS).^21^, ^22^, ^23^, ^24^, ^25^, ^26^, ^27^ The occurrence of irAEs is thought to represent bystander effects from activated T cells and is consistent with the mechanism of ICIs.^28^, ^29^ Another reason to explain why patients experiencing irAEs usually have a better treatment response is that patients with durable response, which means greater ICI exposure, are more likely to develop treatment‐related adverse events. There is less possibility for patients who develop progressive disease soon after ICI initiation to develop irAEs in a short period.

Our results did not reveal the safety concerns of ICIs in special populations. We focused on patients >75 years old, and there was no significant difference in the incidence of irAEs. Several retrospective studies have also attempted to study elderly patients.^30^, ^31^, ^32^, ^33^, ^34^ Only one study reported an increased rate of immune‐related colitis in patients >80 years of age.^34^ Regarding patients with poor ECOG PS (2–3), no evidence showed an inferior safety profile compared to patients with ECOG PS 0–1, though it is still suggested that ICIs should be used cautiously in such patients because of the poor survival benefit and heavy financial burden.^35^ Moreover, we focused on patients with comorbid autoimmune diseases (AID), which is also insignificant in irAE incidence. A retrospective real‐world study was recently conducted on 751 patients with advanced solid tumors treated with anti‐PD‐1 antibodies which found that although the incidence of any grade irAEs was higher in patients with pre‐existing AIDs, no significant difference was observed regarding grade 3–4 irAEs.^36^ No increased toxicity was reported in chronic hepatitis B or hepatitis C infection patients both in our study and in the literature although the evidence in the literature is quite scant.^37^

This study has limitations inherent to its retrospective study design. In this study, we mainly focused on real‐world irAE incidence and spectrum. Therefore, to record as many irAEs, we did not exclude patients whose medical records were not sufficient, which, for example, caused the lack of response assessment. Moreover, we did not include the survival data of patients, which is a drawback in demonstrating the relationship between irAEs and ICI efficacy. In addition, we focused on a special population in a real‐world setting. However, each population sample was small, which may not reveal the objective status.

In conclusion, this study comprehensively analyzed the clinical features and management of ICI‐associated adverse events in a real‐world setting for advanced lung cancer patients in China. As the use of ICIs continues to increase, irAEs have become an increasingly important component of clinical practice. This study adds new evidence regarding real‐world management practices of irAEs for advanced lung cancer.

## CONFLICT OF INTEREST

All authors declare that there are no conflicts of interest.

## FUNDING

This study is funded by CAMS Innovation Fund for Medical Sciences (to MZW) (No: 2018‐I2M‐1‐003).
